# Assessing morphological congruence in *Dinobryon* species and their stomatocysts, including a newly established *Dinobryon pediforme*–stomatocyst connection

**DOI:** 10.1038/s41598-020-65997-9

**Published:** 2020-06-17

**Authors:** Jolanta Piątek, Joanna Lenarczyk, Marcin Piątek

**Affiliations:** 1 Department of Phycology, W. Szafer Institute of Botany, Polish Academy of Sciences, Lubicz 46, PL-31-512, Kraków, Poland; 2 Department of Mycology, W. Szafer Institute of Botany, Polish Academy of Sciences, Lubicz 46, PL-31-512, Kraków, Poland

**Keywords:** Taxonomy, Biodiversity

## Abstract

The chrysophyte genus *Dinobryon* Ehrenberg consists of 44 taxa, which occur in freshwaters, rarely marine waters, mostly in temperate regions of the world. The taxa of *Dinobryon* produce characteristic solitary or dendroid colonies and resting stages called stomatocysts. Only 20 *Dinobryon* taxa have information on produced stomatocysts and only four stomatocysts are reliably linked with vegetative stages using modern identification standards employing scanning electron microscopy (SEM) analyses. In this study, an encysted material of *Dinobryon pediforme* (Lemmermann) Steinecke was collected in two lakes in contrasting regions of Poland. Light microscopy (LM) and scanning electron microscopy (SEM) analyses revealed that *Dinobryon pediforme* produces stomatocyst #61, Piątek J. that is described here as new morphotype following the International Statospore Working Group (ISWG) guidelines. This raises to five the number of reliable links between vegetative stages of *Dinobryon* species and corresponding stomatocysts. Phenotypic similarities between *Dinobryon* species and their stomatocysts, analysed for five reliably established links, showed no relationships in size and shape between loricas and stomatocysts belonging to the same species. The morphological characters of loricas and stomatocysts mapped onto the phylogenetic tree of the five *Dinobryon* species revealed only little congruence between their morphology and phylogenetic relationships.

## Introduction

*Dinobryon* Ehrenberg is one of the most common and recognizable genera of the chrysophytes that are class of heterokont algae belonging to stramenopiles and whose members form resting stages called stomatocysts or cysts. The genus comprises 44 taxa^1–4^, which occur in oligotrophic freshwaters, rarely marine waters, mostly in temperate regions, rarely in arctic and tropical regions of the world. The species of *Dinobryon* form solitary or dendroid colonies with flagella cells inside each lorica. Loricas are usually cylindrical, conical, vase-shaped or funnel-shaped^1^.

As other chrysophytes, *Dinobryon* species have ability to produce siliceous resting stages called stomatocysts, which are formed endogenously within special membrane chambers. Encystment process may individually or collectively depend on several environmental factors, principally the population density of vegetative cells, but also on temperature, nutrient concentrations and endogenous factors^5^. Stomatocysts range from 2 to 30 μm in diameter. They are delineated based on morphology, ornamentation, and height and diameter of the cyst body, collar and pore^6,7^. They are species-specific and classified independently of the vegetative stages. Only 10–15% of the stomatocysts are conclusively linked with living chrysophytes which produce them^8–21^. It is caused by the problems with findings of encysting chrysophytes in nature and cultures; additionally many chrysophytes are usually uncultivable^22^.

Amongst 44 taxa belonging to the genus *Dinobryon*^1–4^ only less than half of them (20 taxa) have information (mostly of limited value) on produced stomatocyst^7,19,21^. For most of these stomatocysts only short description is given complemented with line drawings [12 stomatocysts, produced by *D. annulatum* D.K. Hilliard & Asmund, *D. asmundiae* Nygaard, *D. bavaricum* O.E. Imhof, *D. borgei* Lemmermann, *D. crenulatum* West & G.S. West, *D. crenulatum* f. *callosum* Nygaard, *D. faculiferum* (Willén) Willén, *D. hilliardii* Nygaard, *D. korshikovii* Matvienko, *D. lindegaardii* Nygaard, *D. pediforme* (Lemmermann) Steinecke and *D. sociale* (Ehrenberg) Ehrenberg] or light micrographs (one stomatocyst, produced by *D. unguentariforme* Croome, H.U. Ling & P.A. Tyler). The other three cysts have either only description [stomatocyst of *D. cylindricum* var. *alpinum* (O.E. Imhof) H. Bachmann], or weak SEM micrographs unsuitable for identification [stomatocysts of *D. cylindricum* var. *palustris* Lemmermann and *D. sociale* var. *stipitatum* (Stein) Lemmermann]. The accurate identification of these 16 stomatocysts of *Dinobryon* taxa is therefore impossible considering modern standards necessary for stomatocyst identification, i.e. employing SEM analyses. Only 4 out of 20 stomatocysts linked with vegetative stages of *Dinobryon* taxa are reliably identified using these standards. These are: stomatocysts produced by *D. cylindricum* O.E. Imhof, *D. divergens* O.E. Imhof, *D. sertularia* Ehrenberg and *D. sociale* var. *americanum* (Brunnthaler) H. Bachmann^7,19,21^.

One of the *Dinobryon* species for which encystment process and resultant stomatocyst have been insufficiently characterised before is *Dinobryon pediforme*^7,23^. The encysted populations of this chrysophyte were found in two contrasting regions of Poland. In this study, we aimed to establish a further reliable link between a stomatocyst and a vegetative *Dinobryon* stage, by providing LM- and SEM-based morphological characterization of *D. pediforme* loricas and its stomatocysts. We also tried to assess phenotypic congruence between *Dinobryon* vegetative stages and stomatocysts. For this purpose only those five *Dinobryon* species (*D. cylindricum*, *D. divergens*, *D. pediforme*, *D. sertularia*, *D. sociale* var. *americanum*), for which stomatocysts are reliably characterised using modern standard methods, were taken into account. The morphological characters were mapped onto the phylogenetic tree to analyse the congruence between phylogeny and morphology in chrysophyte species and their stomatocysts.

## Materials and methods

The encysted material of *Dinobryon pediforme* was collected by the second author of this study in two contrasting regions of Poland: from Suchar II lake in the Wigry National Park (north-eastern Poland) on 2^nd^ July 2008 and from Wielki Staw lake in the Karkonosze National Park (south-western Poland) on 29^th^ July 2008. Samples were taken from both lakes using a no. 25 plankton net, either from shore near a hiking trail in Suchar II lake or from shore near the outflow of Biały Potok brook in Wielki Staw lake.

Water temperature, pH and conductivity of Suchar II lake and Wielki Staw lake were measured immediately after material collection with an Elmetron CPC-401 pH/conductivity meter. Other water parameters, including total and carbonate hardness, concentration of oxygen and chloride ions were analysed by titrimetric methods using an Aquamerck Compact Laboratory (Merck) and a Chloride test (Merck).

The examination and identification of *Dinobryon* and stomatocyst specimens were made using standard light and phase contrast microscopy (LM) and scanning electron microscopy (SEM). LM studies were conducted from slide preparations mounted in water, using a Nikon Eclipse 80i light microscope. Micrographs were taken with a Nikon DS-Fi1 camera. For SEM analyses, two methods of material preparation were used. First, fresh material was pipetted onto clean cover glasses, air-dried and affixed to aluminium stubs with a double-sided transparent tape. The stubs were then sputter-coated with carbon using a Cressington sputter-coater and viewed with a Hitachi S-4700 scanning electron microscope. However, in such prepared materials the membrane chamber stuck hard to the stomatocysts, so they were covered, making SEM study difficult. Therefore, for detailed identification of stomatocysts (collar, pore morphology and ornamentation) it was necessary to get rid of the membrane chamber to observe naked specimens. For this purpose material was prepared in the laboratory, where samples were placed in a glasses scintillation vials, covered with 10% HCl and allowed to stand for 24 h. They were then boiled for 15 min, rinsed several times with distilled water, pour in glass vials and covered with 95% alcohol. Finally, such prepared materials were also sputter-coated with carbon and viewed with the microscope mentioned above.

**Figure 1:**
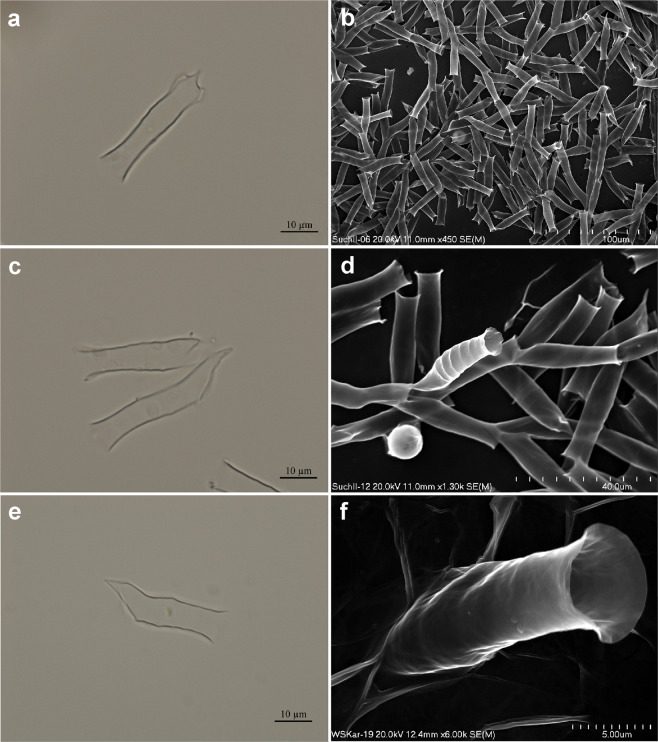
*Dinobryon pediforme* in LM (**a**,**c**,**e**) and in SEM (**b**,**d**,**f**). (**a–d**) *Dinobryon pediforme* observed in Suchar II lake; (**e–f**) *Dinobryon pediforme* found in Wielki Staw lake.

**Figure 2:**
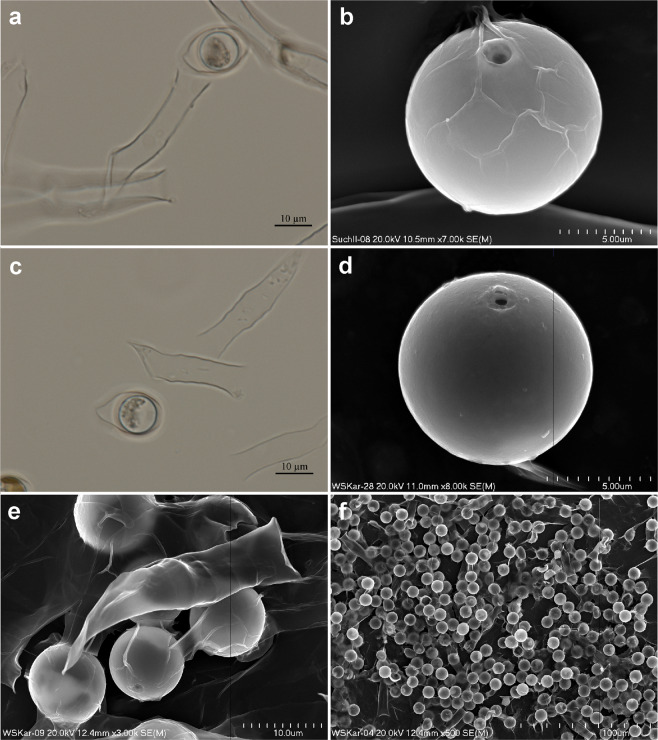
*Dinobryon pediforme* and its stomatocyst #61 in LM (**a**,**c**) and in SEM (**b**,**d**–**f**). (**a**) *Dinobryon pediforme* with stomatocyst #61, which is located within a special encystment chamber, from Suchar II lake; (**b**) Stomatocyst #61 found in Suchar II lake. Note that “ridges” on cyst surface are in fact remnants of encystment chamber; (**c**) *Dinobryon pediforme* with stomatocyst #61, which is located within a special encystment chamber, from Wielki Staw lake; (**d**) Stomatocyst #61 found in Wielki Staw lake; (**e**) One lorica of *Dinobryon pediforme* and stomatocyst #61 from Wielki Staw lake; (**f**) Stomatocyst #61 observed in mass in Wielki Staw lake.

The morphological features of vegetative colonies of *Dinobryon pediforme* found in Suchar II lake and Wielki Staw lake follows the terminology given by Kristiansen & Preisig^1^. Their stomatocyst was characterised according to International Statospore Working Group (ISWG) guidelines^6^, and assigned number #61 Piątek J. to continue the numbering scheme of Piątek^24^. All measurements of *Dinobryon pediforme* loricas and stomatocyst specimens were taken from the fresh material directly under a light microscope and from SEM micrographs. SEM holders are available in the Department of Phycology, W. Szafer Institute of Botany, Polish Academy of Sciences, Lubicz 46, PL-31-512 Kraków, Poland. Morphological characteristics of chrysophyte and stomatocyst are depicted in Figs. 1–2.

Lorica length, lorica width (at the mouth), and stomatocyst diameter in *Dinobryon pediforme* from Suchar II lake and Wielki Staw lake were compared using appropriate statistical tests after testing the assumptions of normality (Shapiro-Wilk test) and equality of variances (*F* test) in Past, ver. 4.01. Lorica length (N = 40) was subjected to the *t* test, lorica width (N = 40) to the Welch test, and stomatocyst diameter (N = 20) to the Mann-Whitney test^25^. *P* values below the threshold 0.05 were treated as statistically significant.

**Figure 3:**
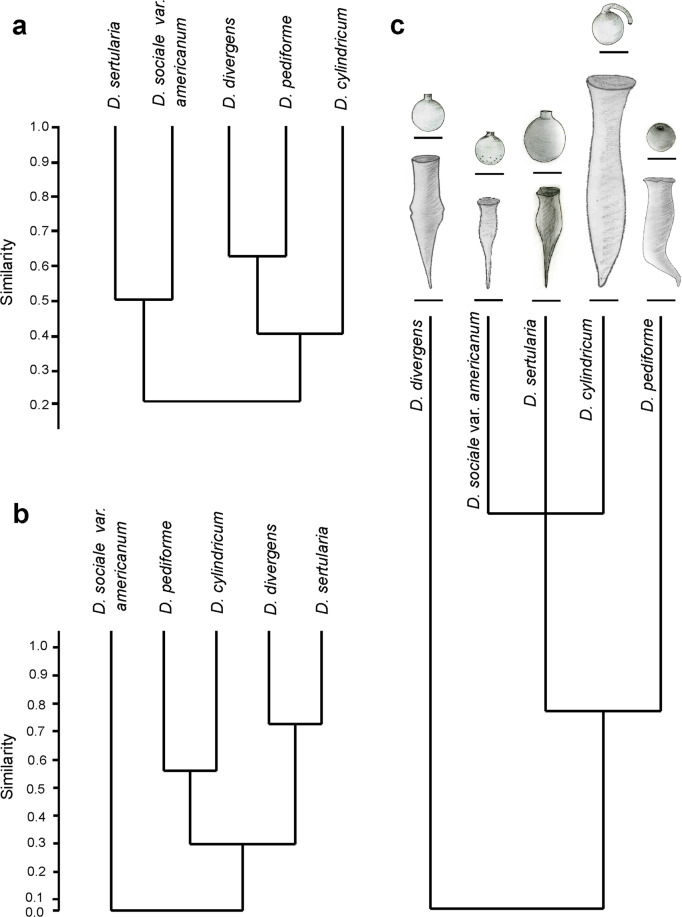
(**a**) Similarities in the shape of lorica between the five *Dinobryon* taxa having stomatocysts assigned to their vegetative stages on the basis of their qualitative characteristics computed using the Jaccard’s coefficient and the UPGMA clustering. Codes of the characteristics are presented in Table 2; (**b**) Similarities in the shape of stomatocyst between the five *Dinobryon* taxa having stomatocysts assigned to their vegetative stages on the basis of their qualitative characteristics computed using the Jaccard’s coefficient and the UPGMA clustering. Codes of the characteristics are presented in Table 2; (**c**) Mapping lorica and stomatocyst morphologies of the five *Dinobryon* taxa having stomatocysts assigned to their vegetative stages on their phylogenetic relationships. The tree is based on the phylogeny of Bock *et al*.^27^. Sizes of the depicted loricas and stomatocysts are means of the sizes included in Table 1.

*Dinobryon pediforme* lorica and stomatocyst newly analysed here, as well as four other *Dinobryon* taxa for which stomatocysts can be reliably assigned (*D. cylindricum*, *D. divergens*, *D. sertularia*, *D. sociale* var*. americanum*, for the sizes and morphological characteristics see Tables 1 and 2), were compared by hierarchical clustering in Past, ver. 3.18, and presented as classification dendrograms (Fig. 3a,b). Clustering was based on the Jaccard’s coefficient (binary similarity index) and the UPGMA method^25^. The occurrence of quantitative morphological characteristics in the classification analysis was marked as “yes”, their absence was marked as “no” (Table 2). Characteristics marked with an asterisk (*) were excluded from the cluster analysis to avoid occurrence common for all five taxa and duplicate values. Both dendrograms were next elaborated using CorelDRAW, ver. 9 (Corel Corporation).

**Table 1:** Sizes of loricas of the *Dinobryon* taxa and respective stomatocysts analysed in this study.

Vegetative stages	*D. cylindricum* O.E. Imhof	*D. divergens* O.E. Imhof	*D. pediforme* (Lemmermann) Steinecke	*D. sertularia* Ehrenberg	*D. sociale* var. *americanum* (Brunnthaler) H. Bachmann
Lenght of lorica	40–115 μm	30–65 μm	27–49 μm	27.5–43.7 μm	24–40 μm
Width of lorica	8–15 μm	8–11 μm	5.0–8.3 μm 5.7–9.2 μm 7.1–11.5 μm	8.0–11.2 μm	7.1–9.6 μm
Source	^36,55^	^36^	this study	^21^	^19^
**Resting stages**	**stomatocyst 41, Duff & Smol 1989**	**stomatocyst 161, Zeeb & Smol 1993**	**stomatocyst #61, Piątek J., this paper**	**stomatocyst 48, Piątek J. 2012**	**stomatocyst 79, Duff & Smol 1991**
Diameter of stomatocyst	9.5–12.6 μm (average 10.8–11.5 μm)	9.6–12.7 μm	9.0–11.4 μm	13.4–16.4 μm	9.0–11.3 μm
Collar diameter of stomatocyst	2.4–4.5 μm	2.1–3.1 μm	basal 1.2–2.9 μm, apical 1.7–2.2 μm	4.2–4.8 μm	2.0–3.1 μm
Collar height of stomatocyst	2.0–3.5 μm	1.0–2.8 μm	0.2–0.5 μm	1.2–1.6 μm	1.4–2.7 μm
Source	^7^	^7^	this study	^21^	^19^

**Table 2:** Qualitative characteristics of the five *Dinobryon* taxa and corresponding stomatocysts analysed in this study.

Vegetative stages		*D. cylindricum* O.E. Imhof	*D. divergens* O.E. Imhof	*D. pediforme* (Lemmermann) Steinecke	*D. sertularia* Ehrenberg	*D. sociale* var. *americanum* (Brunnthaler) H. Bachmann
Colonies	dendroid	no	yes	yes	yes	yes
	scattered	yes	yes	yes	no	no
Lorica body	conical	no	no	no	yes	yes
	*cylindrical	yes	yes	yes	no	no
	middle part distended	yes	no	no	no	no
	middle part wavy	no	yes	no	no	no
Lorica surface	*smooth	yes	yes	yes	yes	yes
	striated	no	no	yes	no	no
Lorica posterior part	short	yes	yes	yes	yes	no
	sharpened	no	yes	yes	no	yes
	oblique	yes	yes	yes	no	no
	1-2 lateral appendages	no	no	yes	no	no
Source		^36^	^36^	this study	^21^	^19^
**Resting stages**		**stomatocyst 41, Duff & Smol 1989**	**stomatocyst 161, Zeeb & Smol 1993**	**stomatocyst #61, Piątek J., this paper**	**stomatocyst 48, Piątek J. 2012**	**stomatocyst 79, Duff & Smol 1991**
Cyst shape	obovate	yes	no	no	no	no
	slightly oval	no	yes	no	no	no
	*spherical	yes	yes	yes	yes	yes
Collar shape	conical	yes	no	yes	no	no
	cylindrical	no	yes	no	yes	no
	*obconical	no	no	no	no	yes
	hooked apex	yes	no	no	no	no
Ornamentation	smooth	yes	yes	yes	yes	no
	scabrae	no	no	no	no	yes
	verrucae	no	no	no	no	yes
Source		^7^	^7^	this study	^21^	^19^

Occurrence of the characteristics was marked as “yes”, their absence as “no”. Characteristics marked with an asterisk (*) were excluded from the cluster analysis to avoid occurrence common for all five taxa and duplicate values.

In order to analyse the level of congruence between morphology and the phylogenetic relationships of the five *Dinobryon* taxa under study, a schematic phylogenetic tree (Fig. 3c) was generated in MorphoJ, ver. 1.06d^26^, and next elaborated using CorelDRAW X4, ver. 9 (Corel Corporation). The tree was based on the evolutionary tree of chrysophytes constructed by maximum likelihood of 18 S rDNA published by Bock *et al*.^27^ where phylogenetic relationships of all the five *Dinobryon* taxa under the present study can be found.

## Results

### Field sampling, and chrysophyte and stomatocyst morphologies

The encysted specimens of *Dinobryon pediforme* originated from natural populations in two freshwater lakes: Suchar II lake and Wielki Staw lake. Suchar II lake (54°05′N, 23°01′E) is a dystrophic lowland lake situated in the Wigry National Park in north-eastern Poland. The lake is surrounded with *Picea abies* forest. Its area is 2.6 ha, maximum depth is 10 m^28^. Wielki Staw lake (50°45′N, 15°42′E) is a subalpine lake situated in the Karkonosze Mountains, in the Karkonosze National Park in south-western Poland. It lies at 1225 m a.s.l. and is the largest water body in these mountains, with the area of 8.3 ha and maximum depth 24.4 m^29^. The occurrence of *Isoëtes lacustris* on the bottom of Wielki Staw lake indicates its low trophy and allows to classify it to lobelia lakes^30^. During the sampling the following physicochemical characters were measured: temperature 21.4 °C and 18.3 °C, pH 5.5 and 5.8, conductivity 17.0 μS cm^−1^ and 11.0 μS cm^−1^, oxygen concentration 7.1 mg l^−1^ and 7.2 mg l^−1^, total and carbonate hardness below 1.0 mmol l^−1^, chloride concentration ca. 6 mg l^−1^ Cl- in Suchar II lake and Wielki Staw lake, respectively.

The abundance of the loricas and stomatocysts differed between both populations. In the sample from Suchar II lake, loricas of *Dinobryon pediforme* occurred massively (Fig. 1a–d), while its stomatocysts occurred rarely, with only single specimens observed in LM and SEM preparations (Fig. 2a–b). The reverse situation was observed in the sample from Wielki Staw lake: loricas of *Dinobryon pediforme* were rare (Fig. 1e–f), but stomatocysts occurred in mass (Fig. 2c–f). In both lakes, mostly empty loricas of *Dinobryon pediforme* without a vegetative cell inside were observed. The stomatocysts occurred separately, but usually within the encystment chambers; however, the stomatocysts included within chambers in the stage of leaving the chrysophyte lorica were also observed (Fig. 2a), confirming their link to *Dinobryon pediforme*. The stomatocyst of *Dinobryon pediforme* was not described before by modern standard methods and is therefore assigned to new morphotype.

The morphologies of *Dinobryon pediforme* and its newly affiliated stomatocyst, based on collected specimens, are given below.

***Dinobryon pediforme*** (Lemmermann) Steinecke Fig. 1a–f.

Synonyms: *Dinobryon protuberans* var. *pediforme* Lemmermann, *D. cylindricum* var. *pediforme* Lemmermann, *D. divergens* var. *pediforme* (Lemmermann) Brunnthaler.

Number of specimens (loricas): in mass in Suchar II lake (Fig. 1a–d), rarely in Wielki Staw lake (Fig. 1e–f).

LM morphology: Colonies dendriod, but mostly disintegrated into single loricas. Lorica bodies cylindrical, hyaline, with smooth or sometimes more or less striated surface; mouth more or less extended; the base asymmetrical with 1–2 lateral processes. Loricas 27.1–49.0 μm long, and 5.7–7.6 μm, 5.7–7.6 μm, 7.1–10.5 μm wide (posterior, middle and anterior parts, respectively).

SEM morphology: Loricas in the anterior part cylindrical, straight or more or less wavy; mouth more or less extended; the posterior asymmetrical with 1–2 lateral processes, short with truncate cone (Fig. 1a). Loricas (29.3–)32.0–46.7 μm long and 5.0–8.3, 6.7–9.2, 8.0–11.5 μm wide (posterior, middle and anterior parts, respectively).

Comments: The sizes of loricas within and between the populations of *Dinobryon pediforme* from Suchar II lake and Wielki Staw lake were roughly similar (length 32.0–49.0 μm and width 5.0–10.0 μm, length 27.1–40.4 μm and width 5.5–11.5 μm, respectively). Statistical differences were observed in lorica length (*t* = 10.91, *p* = 2.34 10^−17^), but not in lorical width (*t* = 0.06, *p* = 0.96), between the two populations. Loricas were smooth and striated even within the same colony in both populations; sometimes, from one parental lorica two morphologically different daughter loricas were emerged: one smooth and other clearly striated (Fig. 1d).

**Stomatocyst #61**, Piątek J., this paper Fig. 2a–f.

Biological affinity: *Dinobryon pediforme* (Lemmermann) Steinecke (established here).

Picture-file number: SuchII–02, Fig. 2b.

Number of specimens: about 30–40 in Suchar II lake (Fig. 2a,b), in mass in Wielki Staw lake (Fig. 2c–f).

LM morphology: Stomatocysts smooth and spherical, 9.2–11.4 μm in diameter, usually 10.5 μm, located within an encystment chambers, pore not visible (Fig. 2a,c).

SEM morphology: Stomatocysts smooth and spherical, 9.0–11.2 μm in diameter, sometimes located within an encystment chambers, which stuck to the cyst wall during the drying SEM preparations (Fig. 2b,e). Collar conical, sometimes with flat planar annulus. Collar in the basal part 1.2–2.9 μm and in the apical part 1.7–2.2 μm in diameter, and 0.2–0.5 μm high. Pore 0.4–0.9 μm in diameter, surrounded by a swollen pseudoannulus or rarely by a planar pseudoannulus.

Comments: The sizes of stomatocysts, and their collars and pores in two studied populations were roughly similar. The cyst diameter was 9.5–11.4 μm in Suchar II lake and 9.0–10.5 μm in Wielki Staw lake, but differed statistically (*U* = 32, *p* = 5.54 10^−6^). The collar of stomatocysts had the same height in both populations, but in the population from Wielki Staw lake the collar had an irregular edge. Another difference was observed in the size of basal diameter of the collar, which was smaller in the population from Wielki Staw lake (1.2–2.0 μm) than in the population from Suchar II lake (2.1–2.9 μm). Finally, some differences were also observed in the size of the pore diameter, which was smaller in specimens from Wielki Staw lake (0.4–0.7 μm) than from Suchar II lake (0.7–0.9 μm).

Stomatocyst #61, Piątek J. belongs to a series of stomatocysts having similar morphologies but different cyst body sizes, including stomatocyst 52, Duff & Smol 1991 *emend*. Duff *et al*.^7,31^; stomatocyst 234, Duff *et al*.^7^; stomatocyst 127, Duff & Smol in Duff *et al*.^32^; and stomatocyst 197, Duff & Smol 1994^33^. According to Duff *et al*.^7^, the ranges of cyst body sizes for these stomatocysts are <5 μm, 5.0–9.9 μm, 10.0–15.0 μm, and > 15.0 μm, respectively. In such scheme, the stomatocyst of *Dinobryon pediforme* is placed between the upper size values of stomatocyst 234 and lower size values of stomatocyst 127. The original cyst body sizes reported for the latter cyst were 12.6–15.0 μm^32^ and only later augmented to 10.0–15.0 μm^7^. Considering this fact, the stomatocyst of *Dinobryon pediforme*, described here as new stomatocyst #61, Piątek J., perfectly fits into the “space” between sizes of stomatocyst 234 and stomatocyst 127. Consequently, the following ranges of cyst body sizes for the discussed group of stomatocysts are newly delineated here (some values were rounded): stomatocyst 52 <5 μm, stomatocyst 234 – 5.0–9.0 μm, stomatocyst 61–9.0–12.0 μm, stomatocyst 127–12.0–15.0 μm, and stomatocyst 197 >15.0 μm.

### Comparative analysis of phenotypic congruence between *Dinobryon* vegetative stages and their stomatocysts

Comparative analysis of lorica morphology of five *Dinobryon* taxa, whose stomatocysts are reliably characterised (*D. cylindricum*, *D. divergens*, *D. pediforme*, *D. sertularia*, *D. sociale* var*. americanum*), revealed that *D. pediforme*, *D. sertularia* and *D. sociale* var*. americanum* produce shorter loricas than *D. cylindricum* and *D. divergens*, but all of them have similar width (with the exception of *D. cylindricum*, which lorica is somewhat wider) (Table 1).

The shape of vegetative stages of *Dinobryon pediforme* was most similar to the stages of *D. divergens*. Both taxa produce dendroid or scattered colonies composed of cylindrical loricas with short, sharpened and oblique ends. The other taxon, *D. cylindricum*, producing scattered colonies of cylindrical loricas with a short, oblique, but not sharpened ends, is similar to these two taxa. The lorica posterior part in *D. pediforme* can posses additional 1–2 lateral appendages, whereas *D. divergens* has a wavy middle part of lorica and *D. cylindricum* has a distended one. In contrast, the colonies of *D. sertularia* and *D. sociale* var. *americanum* are dendroid and composed of conical loricas with a straight posterior part (Fig. 3a,c, Table 2).

No relationships in size between loricas and stomatocysts belonging to the same taxa were observed. Four of five taxa under study with different sizes of lorica produce stomatocysts which are similar in size. *Dinobryon sertularia*, whose loricas are rather short, produces clearly bigger stomatocysts. In three taxa (*D. divergens*, *D. pediforme* and *D. sociale* var. *americanum*), their stomatocyst diameter is similar to the lorica width, except for that of *D. sertularia* which produces cysts much bigger than the lorica width (Table 1).

The collar diameter is bigger that its height in both *Dinobryon pediforme* and *D. sertularia*. Both species produce stomatocysts with a short collar, compared to the other three taxa. The collar diameter and the collar height overlap in the remaining three taxa (Table 1).

The shape of stomatocysts is not connected with the shape of vegetative stages which produce them, as taxa most similar to each other with respect to shape of loricas are not similar to each other with respect to the shape of stomatocysts. *Dinobryon sertularia* forms a common clade either with *D. sociale* var. *americanum* in the tree showing the shape of loricas or with *Dinobryon divergens* in the tree showing the shape of stomatocysts (Fig. 3a–b, Table 2).

The stomatocyts of *Dinobryon divergens* and *D. sertularia*, mostly similar with regard to their shape, are spherical or slightly oval (in *D. divergens*), smooth and have a cylindrical collar. The stomatocysts of *D. pediforme* and *D. cylindricum* are also very similar to each other. They are spherical and smooth, but have conical collars, sometimes with an irregular edge. *Dinobryon cylindricum* produces stomatocysts, which are sphaerical or obovate, smooth and have conical collars with a hooked apex. In contrast, stomatocysts of *D. sociale* var. *americanum*, forming a separate lineage in the tree showing the cyst morphologies, are covered with scabrae and verrucae, mainly in the posterior hemisphere, and have obconical collars (Fig. 3b, Table 2).

Mapping morphological data on loricas and stomatocysts of the five analysed *Dinobryon* taxa on the phylogenetic tree shows that their sizes are not connected with phylogeny, whereas their shapes only little reflect natural evolutionary relationships of the taxa. *Dinobryon sociale* var. *americanum*, *D. sertularia* and *D. cylindricum*, being most closely related, produce different types of colonies, loricas and stomatocysts (including their collar and ornamentation). *Dinobryon divergens*, which forms a separate phylogenetic lineage, possesses wavy middle parts of loricas, which is a clear difference between this taxon and the remaining four taxa (Fig. 3c, Tables 1 and 2).

## Discussion

*Dinobryon pediforme* is a freshwater species, occurring in subarctic regions and humic acid waters^34^. It is recorded from Europe and North America, and it was recently used as a bioindicator species tied with strongly acid, often humic conditions^35^. In this study, the encystment of *Dinobryon pediforme* was observed in two natural populations in Poland. It is worth to note that the encystment process of chrysophytes in natural populations is rarely observed^5^ and usually only a small fraction of living chrysophyte cells undergoes the stomatocyst development^13,14^. Previously, the stomatocyst production by *Dinobryon pediforme* was documented only few times by line drawings from observations made in light microscope. The illustrative documentation was, however, weak and contradictory. Krieger^23^ (repeated by Starmach^36,37^), Nygaard^38^ and Juriš^39^ depicted globoid stomatocysts with a pore lacking collar. Stomatocysts were located in special encystment chambers and were in stage of leaving the lorica. The pore of stomatocysts was oriented towards the upper part of encystment chamber. By contrast, Skuja^40^ and Ermolaev and Safonova^41^ depicted globoid stomatocysts with a well developed collar (and therefore invisible pore). Stomatocysts were located in special encystment chambers and were in stage of leaving the loricas, but the collar (with invisible pore) of stomatocysts was oriented towards the lower part of the chamber. The morphology of stomatocysts of *Dinobryon pediforme* observed in Poland were more similar to those depicted by Krieger^23^, Nygaard^38^ and Juriš^39^ than to those illustrated by Skuja^40^ and Ermolaev and Safonova^41^. The collar and pore were not visible in LM, but the low collar and pore were visible in SEM. The stomatocyst produced by *Dinobryon pediforme* cannot be assigned to any of the stomatocysts described by modern SEM-based methods and is therefore described in this study as new morphotype.

With this study, the number of reliably established *Dinobryon*-stomatocyst links raised to five and include *D. cylindricum*–stomatocyst 41, Duff & Smol 1989^7,42^, *D. divergens*–stomatocyst 161, Zeeb & Smol 1993^7,43^, *D. pediforme*–stomatocyst #61, Piątek J. (this study), *D. sertularia*–stomatocyst 48, Piątek J. 2012^21^, and *D. sociale* var*. americanum*–stomatocyst 79, Duff & Smol 1991^19,31^. All these *Dinobryon* taxa are freshwater species. Four of them (*D. cylindricum*, *D. divergens*, *D. sertularia*, *D. sociale* var. *americanum*) are common cosmopolitan species, recorded frequently from different localities worldwide. *Dinobryon pediforme* has been recorded so far only from Europe and North America. The known geographical distribution of these *Dinobryon* species generally corresponds to the known distribution of their stomatocysts. Species of *Dinobryon* were recorded more frequently and from more continents because they were longer studied and by more researchers compared to stomatocysts. A good example might be the case of *D. sertularia*, which was recorded many times from all continents except Antarctica, while its stomatocyst – stomatocyst 48, Piątek J. 2012^21^ was recorded only from Africa.

A common view is that stomatocysts are species-specific^10,16^. The same holds true for the analysed *Dinobryon* taxa – all of them have specific stomatocysts. An interesting case is *Dinobryon cylindricum*. Its cyst is assigned to stomatocyst 41, Duff & Smol 1989^7,42^. Two other cysts, stomatocysts 78, Duff & Smol 1991^31^ and 233, Zeeb *et al*.^44^ are similar morphologically and could be produced by closely related *Dinobryon* species^7^. It is also not excluded that they all are produced by *Dinobryon cylindricum* but represent three developmental phases: immature stomatocyst, slightly immature stomatocyst and mature stomatocyst^14,45^.

Other than linking *Dinobryon pediforme* with new stomatocyst #61, Piątek J., this study assessed phenotypic congruence between vegetative stages of selected *Dinobryon* taxa (*D. cylindricum*, *D. divergens*, *D. pediforme*, *D. sertularia* and *D. sociale* var. *americanum*) and their stomatocysts, as well as mapping respective morphologies onto phylogenetic tree.

No relationships in size between loricas and stomatocysts belonging to the same taxa analysed in the present study may result from different amounts of elements which are used by individual taxa to build stomatocysts. So far, we do not have much data on environmental conditions causing the encystment process^5^, as many chrysophyte taxa are usually not willing to grow in cultures and finding the encysting specimens in natural and laboratory conditions is problematic^22^. The fact that the diameter of stomatocysts in analysed *Dinobryon* species was similar or bigger (in *D. sertularia*) than the lorica width resulted from the encystment process which in all *Dinobryon* species occurs within the special chamber at the mouth of the lorica^1^. This character may be, however, genetically fixed.

Similarly, the shape of stomatocysts of the five *Dinobryon* taxa analysed in the present study is not connected with the shape of vegetative stages which produce them. This means that the morphological evolution of loricas goes independently from that of stomatocysts. However, some genes can be linked and, consequently, responsible for common occurrence of some morphological traits, which can be observed in both plant and animals^46^.

The genomic and phylogenetic studies on the genus *Dinobryon* are still at the initial level^20,22,27,47–50^. The comprehensive studies of Bock *et al*.^27^, whose 18S rDNA phylogeny was a reference for the tree reconstructed in the present study, analysed relationships between all five taxa being under the present study and additional two taxa, *D. sociale* and *D*. cf. *sociale*. Only little congruence between morphology of the five *Dinobryon* taxa and their phylogeny revealed in the present study suggests that morphological characters can be the effect of homoplasy or DNA regions used for reconstructing phylogenetic relationships so far are not sufficient. Such lack of congruence has previously been found in many microscopic algae^51–53^. However, congruence between morphology and molecular phylogeny has been observed in the chrysophyte genus *Synura*^54^. Mapping morphological traits on the tree based on the whole nuclear genomes would be more reliable and must be the aim of future research. Together with matching other *Dinobryon* taxa with their stomatocysts, genomic studies should be the next necessary step to understand the phylogeny of this genus.
